# Current Insights into Obesity and m^6^A Modification

**DOI:** 10.3390/biomedicines13092164

**Published:** 2025-09-05

**Authors:** Chen Meng, Di Yang

**Affiliations:** Human Phenome Institute, Center for Medical Research and Innovation, Shanghai Pudong Hospital, Fudan University Pudong Medical Centre, Zhangjiang Fudan International Innovation Center, Fudan University, Shanghai 201203, China; mengchen2347@163.com

**Keywords:** obesity, m^6^A modification, methylation, adipogenesis, epigenetics, metabolic diseases, therapeutic targets

## Abstract

Obesity has emerged as a global health challenge, closely associated with multiple metabolic diseases, such as cardiovascular diseases, type 2 diabetes, and non-alcoholic fatty liver disease. The traditional “calories-in minus calories-out” paradigm is no longer sufficient to explain the heterogeneity of obesity; consequently, a growing body of research has turned its focus to epigenetic regulation—particularly chemical modifications at the RNA level. *N*^6^-methyladenosine (m^6^A) modification is one of the most abundant epigenetic modifications on RNA, which dynamically regulates the methylation reaction in specific sequences on mRNA through methyltransferases (writers), demethylases (erasers), and binding proteins (readers). Accumulating evidence in recent years has revealed that m^6^A modification plays a pivotal role in the pathogenesis and progression of obesity, particularly through its regulation of key biological processes, such as adipocyte differentiation, lipid metabolism, and energy homeostasis. Given its critical involvement in metabolic dysregulation, targeting m^6^A-related mechanisms may offer novel therapeutic avenues for obesity management. This review systematically summarizes the current understanding of m^6^A modification in obesity, elucidates its underlying molecular mechanisms, and evaluates its potential as a therapeutic target. By integrating recent advances in the field, we aim to provide new perspectives for the development of innovative strategies in obesity treatment.

## 1. Introduction

Obesity is a complex metabolic disorder characterized by excessive adipose tissue accumulation and dysfunctional fat distribution, which induces chronic low-grade systemic inflammation and metabolic dysregulation, including dyslipidemia, hyperglycemia, and hyperuricemia. Epidemiological and clinical studies have established a strong association between obesity and multiple metabolic disorders, such as cardiovascular diseases (CVD), type 2 diabetes mellitus (T2DM), hypertension, and non-alcoholic fatty liver disease (NAFLD) [1,2]. Furthermore, obesity serves as a significant risk factor for comorbid conditions, including obstructive sleep apnea (OSA) and osteoarthritis (OA), exacerbating their pathogenesis and clinical severity [3,4]. Beyond its pathophysiological consequences, obesity imposes a substantial burden on global public health, severely compromising patients’ quality of life. Alarmingly, obesity contributes to an estimated 4 million deaths worldwide per year, with its prevalence exhibiting a persistent upward trend [5]. Therefore, it has become increasingly urgent to find more effective strategies to combat obesity.

Epigenetics investigates the regulation and transmission of genetic information without alterations to the underlying DNA sequence. It explores the mechanisms of gene expression regulation and how external environmental factors influence gene expression. The key mechanisms of epigenetics mainly include DNA methylation, post-transcriptional modifications, and histone modifications [6]. Among these, post-transcriptional modifications play a crucial role in various physiological processes. Recent studies revealed diverse internal modifications within eukaryotic mRNA, including *N*^6^-methyladenosine (m^6^A), additional methylations of adenosine to form N1-methyladenosine (m^1^A) and *N*^6^,2′-O-dimethyladenosine (m^6^Am), as well as cytosine methylation to 5-methylcytosine and its oxidation product 5-hydroxymethylcytosine (hm^5^C). m^6^A is the most common modification in the mammalian RNA transcriptome, which is essential for modulating gene expression, RNA stability, splicing, and translation. Initially identified in 1974, m^6^A modification remained a relatively underexplored field for decades. However, advances in next-generation sequencing (NGS)-based m^6^A detection methods have propelled significant breakthroughs over the past decade, laying a solid foundation for a deeper understanding of its biological functions [7]. m^6^A modification is highly conserved in the mRNA of most eukaryotes and viruses and is widely present in almost all types of RNA, spanning the entire RNA lifecycle [8]. m^6^A modification sites are primarily enriched near the stop codon and the 3′ untranslated region (3′ UTR) and exhibit a conserved RRACH (R = G/A, H = A/C/U) sequence motif [9,10]. m^6^A modification has been confirmed as a key regulatory factor in the progression of various diseases, positioning it as a major research focus in biomedical science. This paper focuses on the potential link between m^6^A modification and obesity, aiming to provide novel perspectives and mechanistic insights to advance research in this field.

## 2. Obesity

In mammals, the development of obesity primarily arises from a dysregulation of energy balance, characterized by an imbalance between energy intake, expenditure, and conversion [11]. The physiological system that maintains energy homeostasis achieves balance through the fine regulation of energy intake and expenditure. The hypothalamus plays a central role in this regulatory process, integrating and processing complex metabolic signals. This system responds not only to long-term hormonal regulators, such as leptin and insulin, but also to short-term satiety and nutrient-sensing cues. Ultimately, hypothalamic output modulates energy balance by orchestrating appetite control, energy expenditure, circulating hormone levels, and physical activity [12].

Physiologically, obesity develops primarily through adipocyte hyperplasia (increase in cell number) and hypertrophy (increase in cell size). During infancy and adolescence, adipogenesis leads to the continuous expansion of adipocyte populations. In adulthood, adipocyte turnover maintains a relatively stable cell number through an annual renewal rate of approximately 10% [13,14]. Consequently, adult-onset obesity predominantly results from adipocyte hypertrophy rather than hyperplasia. Adipose tissue can be classified into white adipose tissue (WAT), brown adipose tissue (BAT), and beige adipose tissue, based on differences in cell morphology, function, and metabolic characteristics. WAT is mainly composed of large, spherical fat cells packed closely together and primarily serves as a store for chemical energy. BAT contains multilocular adipocytes rich in mitochondria that express uncoupling protein 1 (UCP1), functioning as a thermogenic organ. Beige adipose tissue, located primarily in subcutaneous WAT depots (e.g., inguinal region), represents a thermogenically inducible population. When exposed to stimuli such as cold exposure, beige adipose tissue undergoes browning—characterized by mitochondrial biogenesis, multilocular lipid droplet formation, and robust UCP1 expression—acquiring BAT-like characteristics [15].

Adipocytes originate from multipotent mesenchymal stem cells (MSCs), which possess the pluripotent capacity to differentiate into various cell types, including adipocytes, muscle cells, chondrocytes, and osteocytes. Adipogenesis occurs through two distinct but interconnected phases: lineage commitment and terminal differentiation. During lineage commitment, multipotent MSCs are induced to transform into pre-adipocytes, acquiring adipogenic potential while losing competence for alternative lineages. The subsequent differentiation phase involves preadipocyte maturation into functionally competent adipocytes, characterized by lipid accumulation and endocrine function [16,17]. Recent decades have witnessed significant advances in understanding the complex transcriptional hierarchies governing adipogenesis [18]. These networks precisely coordinate the expression of genes essential for adipocyte development, lipid metabolism, and endocrine signaling. Proper regulation of these molecular cascades is critical for maintaining adipose tissue homeostasis, while their dysregulation has been implicated in the pathogenesis of metabolic disorders, particularly obesity.

## 3. m^6^A Modification

**Figure 1:**
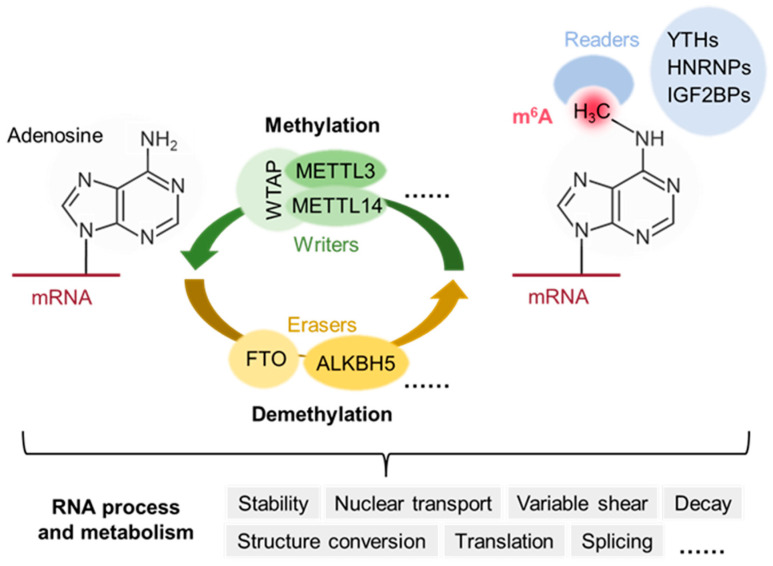
The molecular mechanism and biological functions of m^6^A modification. The adenosine (A) bases in mRNA could be methylated to form *N*^6^-methyladenosine (m^6^A) by the methyltransferase complex (writers). m^6^A could be reversibly removed by demethylases (erasers) or could be recognized by m^6^A binding proteins (readers) to affect mRNA fate.

The m^6^A modification represents a highly dynamic and reversible regulatory process governed by three principal classes of regulatory proteins: the methyltransferase complex (writer), demethylases (eraser), and binding proteins (reader). These factors are responsible for the addition, removal, and recognition of m^6^A modifications on RNA, respectively. Their coordinated action finely tunes the dynamic balance of m^6^A modifications, thereby influencing RNA metabolism and gene expression (Figure 1).

The m^6^A methyltransferase catalyzes site-specific methylation at consensus RNA sequences. In 1994, Bokar and colleagues first discovered that methyltransferases function as a protein complex, providing the foundation for understanding the molecular mechanisms of m^6^A modification [19]. Current research has identified the methyltransferase-like 3 (METTL3) and methyltransferase-like 14 (METTL14) heterodimers as the catalytic core of the m^6^A methyltransferase complex (MTC), where their structural interaction enables cooperative m^6^A deposition [20]. The Wilms tumor 1-associated protein (WTAP) serves as a critical regulatory component that modulates MTC activity through direct physical association with METTL3 [21]. Additionally, factors such as VIRMA, HAKAI, ZC3H13, and RBM15B are involved in the functional regulation of MTC [22,23,24,25]. Apart from MTC, other independent m^6^A methyltransferases, including METTL16, METTL5, and ZCCHC4, also play significant roles in various biological processes [26,27,28].

The m^6^A demethylase family, comprising fat mass and obesity-associated protein (FTO) (first identified in 2011) and AlkB homolog 5 (ALKBH5) (discovered in 2013), plays a pivotal role in RNA metabolism by catalyzing the reversible removal of m^6^A modifications to regulate mRNA processing and nuclear export [29,30]. Recent studies have also shown that ALKBH1 and ALKBH3 exhibit demethylation activity, further expanding the functional scope of the m^6^A demethylase family [31,32]. These α-ketoglutarate-dependent dioxygenases mediate m^6^A demethylation through a conserved catalytic mechanism involving Fe(II)-coordinated molecular oxygen substitution for bound water at their active sites [33], highlighting their shared biochemical properties despite functional diversity in RNA epigenetic regulation.

m^6^A binding proteins specifically recognize m^6^A modification sites, thereby triggering a cascade of downstream biological processes. To date, the m^6^A binding proteins primarily include three classes: YTH domain-containing proteins (YTHs), heterogeneous nuclear ribonucleoproteins (HNRNPs), and the IGF2BP family (IGF2BPs). The YTH protein family consists of YTHDF1 [34], YTHDF2 [35], YTHDF3 [36], YTHDC1 [37], and YTHDC2 [38], all of which are extensively involved in various stages of mRNA metabolism, with each member playing a distinct biological role. HNRNPs are predominantly localized in the nucleus, with key members, such as HNRNPA2/B1 [39], HNRNPC [40], and HNRNPG [41], contributing critically to different stages of mRNA maturation. The IGF2BP family, comprising IGF2BP1, IGF2BP2, and IGF2BP3, enhances mRNA stability and facilitates translation efficiency [42]. In addition, other binding proteins, such as EIF3 [43] and FMRP [44], participate in regulatory processes through direct or indirect interactions, thereby broadening the functional repertoire of m^6^A modifications.

## 4. The Regulatory Role of m^6^A Modification in Adipogenesis

The dynamic interplay between m^6^A writers, erasers, and readers precisely modulates m^6^A deposition patterns on RNA transcripts, thereby post-transcriptionally regulating the expression and metabolic processing of adipogenic factors to control adipocyte differentiation and maturation. This section systematically examines the distinct molecular mechanisms through which each class of m^6^A regulators orchestrates adipogenesis, with particular emphasis on their target specificity and downstream regulatory networks.

### 4.1. m^6^A-Mediated Regulation of Adipocyte Lineage Commitment

During early postnatal development in mice, METTL3 levels significantly increase in interscapular brown adipose tissue, playing an essential role in its development [45]. Similarly, the knockdown of WTAP in brown adipocytes inhibits the differentiation of embryonic stem cells into mature brown adipocytes [46]. Conversely, the deletion of METTL3 in skeletal mesenchymal stem cells leads to an increased accumulation of bone marrow adipose tissue [47,48]. In contrast, the m^6^A demethylase FTO is crucial for promoting adipogenesis. Studies have demonstrated that the GDF11-FTO signaling pathway regulates the differentiation of mouse bone marrow mesenchymal stem cells into adipocytes by targeting PPARγ, a process that is dependent on the m^6^A demethylase activity of FTO [49]. Additionally, FTO was shown to regulate adipogenesis in the early stages of porcine primary preadipocyte differentiation through the JAK2–STAT3–C/EBPβ signaling pathway [50]. In summary, the methyltransferases and demethylases are pivotal in regulating adipocyte lineage commitment and the development of adipose tissue, although their effects are opposite.

### 4.2. m^6^A-Mediated Regulation of Adipocyte Terminal Differentiation

FTO mediates m^6^A demethylation, thereby influencing the RNA metabolism of regulatory factors associated with adipogenesis and playing a crucial role in the terminal differentiation of adipocytes. Specifically, FTO promotes cell differentiation by modulating the splicing of Srsf2 mRNA [51] and is indispensable during the differentiation of 3T3-L1 cells, with its effects mediated through the key adipogenesis regulator PPARγ [52]. Additionally, another demethylase, ALKBH5, impacts adipogenesis by regulating the expression of TRAF4 [53]. YTHDF2 modulates the mRNA stability and expression of ATG5 and ATG7 via an m^6^A -dependent pathway, thereby influencing adipogenesis [54]. Furthermore, YTHDF1 promotes the translation of ADIPOQ through an m^6^A modification-dependent mechanism, thereby enhancing the adipogenesis process in porcine longissimus dorsi muscle [55]. Collectively, m^6^A-related factors finely regulate adipogenesis through various signaling pathways and mechanisms, providing essential insights into the molecular regulatory mechanisms of adipose tissue development.

## 5. The Regulatory Role of m^6^A Modification in Lipid Metabolism

Lipids, including fatty acids, phospholipids, cholesterol, and their derivatives, are essential cellular components that serve as important energy storage molecules, structural constituents of biological membranes, and key players in signal transduction. Numerous studies have highlighted the crucial role of m^6^A modification in regulating lipid metabolism. Pioneering studies in yeast first established the conserved role of m^6^A in lipid metabolism, demonstrating that the methyltransferase IME4 modulates triglyceride metabolism through transcriptional regulation of fatty acyl-CoA synthetase FAA1 [56]. Subsequent investigations in mammalian systems utilizing MeRIP-seq analysis revealed enrichment of m^6^A modifications in lipid metabolic pathways during high-fat diet-induced hepatic steatosis [57], suggesting direct epigenetic control of lipogenic gene networks. Additionally, studies have shown that the m^6^A eraser FTO promotes triglyceride accumulation in HepG2 cells by reducing m^6^A levels, indicating that FTO-mediated demethylation of m^6^A enhances lipid metabolism [58]. Furthermore, knocking down METTL3 or YTHDF2 inhibits lipid accumulation in HepG2 cells [59], collectively demonstrating the bidirectional regulation of lipid metabolism by m^6^A machinery and highlighting its therapeutic potential for metabolic disorders, including non-alcoholic fatty liver disease.

## 6. The Regulatory Role of m^6^A Modification in Regulating Mitochondrial Function

The m^6^A modification plays a pivotal role in regulating mitochondrial function, which is central to diverse physiological processes, including energy metabolism, biosynthesis and degradation of biomolecules, apoptosis, and immune responses. Peroxisome proliferator-activated receptor gamma coactivator 1-alpha (PGC1α) serves as a key regulator of mitochondrial function. In inflammatory monocytes, METTL3 and YTHDF2 collaboratively suppress PGC1α expression, thereby reducing ATP production and oxygen consumption. Additionally, METTL3 knockout was shown to mitigate mitochondrial inflammatory damage induced by oxidized low-density lipoprotein (oxLDL), highlighting the critical role of m^6^A modification in regulating mitochondrial function and the inflammatory response [60]. Docosahexaenoic acid (DHA) promotes aerobic oxidation and mitochondrial biogenesis by upregulating PGC1α expression. Mechanistically, DHA enhances FTO expression, which in turn reduces global m^6^A modification levels. This reduction inhibits YTHDF2-mediated decay of DNA damage-inducible transcript 4 (Ddit4) mRNA. The stability of Ddit4 mRNA influences downstream PGC1α expression, thereby modulating mitochondrial function and energy metabolism [61]. FTO overexpression has been demonstrated to inhibit mitochondrial fission and promote fusion by regulating several factors related to mitochondrial dynamics, leading to decreased mitochondrial content and ATP levels [58]. Furthermore, FTO activates the JAK2/STAT3 signaling pathway to promote lipogenesis and inhibit mitochondrial unfolded protein response-induced apoptosis in adipocytes by reducing m^6^A levels [50]. ALKBH1 was also shown to localize to mitochondria and influence the proliferation of HEK293 and HEK293T cells, although the precise mechanisms remain to be fully elucidated [62].

## 7. The Regulatory Role of m^6^A Modification in Neurological Regulation

**Figure 2:**
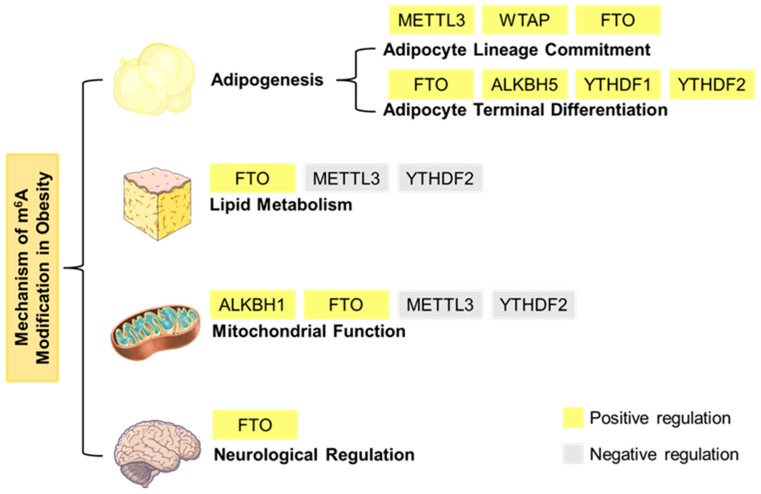
Mechanism of m^6^A modification in obesity. m^6^A modification plays a crucial role in a variety of physiological processes, including adipogenesis, lipid metabolism, mitochondrial function, and neurological regulation.

The hypothalamus serves as a critical control center for food intake and energy homeostasis. FTO is highly expressed in this region, with its expression levels fluctuating under different nutritional conditions, thus being closely linked to the regulation of systemic metabolism. Studies have demonstrated that Fe^2+^ weakens the binding of FTO to its own promoter, thereby enhancing its gene expression and forming an autoregulatory loop involved in the hypothalamic control of body weight [63]. Furthermore, elevated hypothalamic expression of FTO and CX3CL1, coupled with increased levels of the cytokine signaling suppressor SOCS3, disrupts leptin signaling, promoting leptin resistance and obesity [64]. Additionally, high FTO expression increases the abundance of growth hormone-releasing peptide (GHRP) mRNA while specifically reducing its m^6^A modification levels. This alteration results in increased energy intake, ultimately contributing to the development of obesity [65]. Collectively, these findings highlight the role of m^6^A modification in modulating key hypothalamic appetite-regulatory genes, thereby influencing energy balance and contributing to obesity pathogenesis. However, the precise mechanisms underlying these regulatory processes remain to be fully elucidated (Figure 2).

## 8. The Regulatory Role of m^6^A Modification in Obesity-Related Diseases

### 8.1. The Role of m^6^A Modification in Type 2 Diabetes

Obesity is closely associated with insulin secretion dysfunction and impaired insulin sensitivity, significantly increasing the risk of type 2 diabetes in both rodents and humans. Recent studies have underscored the critical role of m^6^A modification in regulating insulin secretion and sensitivity. Mice with a brown adipose tissue (BAT)-specific knockout of the METTL14 gene exhibit enhanced insulin sensitivity and glucose tolerance, independent of body weight, gender, or classical BAT thermogenesis [66]. Pancreatic β-cell-specific METTL14 knockout mice display significantly reduced m^6^A modification levels in the islets, along with decreased β-cell proliferation and an early onset of diabetes, closely resembling the islet dysfunction observed in human type 2 diabetes [67]. Additionally, liver-specific knockdown of METTL3 was shown to decrease the m^6^A methylation of fatty acid synthase (FASN), consequently ameliorating high-fat diet-induced insulin resistance in murine models [59]. These findings highlight the vital role of m^6^A modification in regulating insulin secretion and sensitivity, positioning m^6^A-related regulatory machinery as promising therapeutic targets for obesity-related type 2 diabetes.

### 8.2. The Role of m^6^A Modification in Non-Alcoholic Fatty Liver Disease

Non-alcoholic fatty liver disease (NAFLD) is a complex metabolic disorder characterized by pathological lipid accumulation in hepatocytes, independent of alcohol consumption. Hepatic steatosis, a hallmark of NAFLD, primarily results from dysregulation in metabolic processes such as de novo lipogenesis, impaired fatty acid β-oxidation, altered lipid uptake, and defective triglyceride export mechanisms [68]. Emerging evidence implicates FTO as a critical regulator of hepatic lipid metabolism, where its activity in reducing m^6^A modification levels promotes intracellular triglyceride deposition and contributes to hepatic steatosis [58]. Elevated FTO levels in the liver contribute to lipid accumulation and accelerate the progression of NAFLD [69]. Notably, the natural compound curcumin has shown therapeutic potential by upregulating key methyltransferases METTL3 and METTL14, thereby mitigating lipopolysaccharide-induced hepatic injury and restoring normal lipid metabolism patterns [70]. Additionally, it has been demonstrated that the natural polyphenolic compound resveratrol can ameliorate the disruption of hepatic lipid homeostasis induced by a high-fat diet by reducing m^6^A modification levels [71]. These findings collectively establish m^6^A RNA modification as a multifaceted regulator influencing both the initiation and progression of NAFLD through diverse molecular mechanisms affecting hepatic lipid homeostasis.

### 8.3. The Role of m^6^A Modification in Cardiovascular Diseases

Recent studies have highlighted the crucial role of m^6^A modification in the pathological processes of various cardiovascular diseases, encompassing cardiac hypertrophy, heart failure, ischemic heart disease, aortic aneurysm, vascular calcification, and pulmonary hypertension. During cardiac remodeling, m^6^A methylation levels in cardiomyocytes significantly increase in response to hypertrophic stimuli. Experiments evidence that cardiomyocyte-specific overexpression of METTL3 can induce compensatory hypertrophic responses [72]. The expression of ALKBH5 increased in hypertrophic heart [73]. Conversely, knockdown of FTO in cardiomyocytes substantially increases arrhythmia susceptibility, suggesting that FTO is essential in the onset and progression of heart failure [74]. Macrophage-specific knockout of ALKBH5 was shown to inhibit Angiotensin II (Ang II)-induced macrophage-to-myofibroblast transition, thereby improving cardiac fibrosis and dysfunction [75]. Abdominal aortic aneurysm (AAA) is a common vascular disease with a high mortality rate. Compared to healthy aortic tissue, m^6^A modification levels are significantly elevated in AAA. METTL14 has been implicated in inflammatory infiltration and neovascularization in AAA [76]. Additionally, METTL14 also contributes critically to vascular calcification pathogenesis, as evidenced by its upregulated expression in calcified arterial tissues and associated global increase in m^6^A methylation [77]. Furthermore, m^6^A modification participates in pulmonary hypertension pathogenesis by modulating circRNA stability within the circRNA–miRNA–mRNA regulatory network, ultimately activating Wnt and FoxO signaling pathways to promote disease onset [78]. These collective findings underscore the multifaceted involvement of m^6^A modification in cardiovascular disease pathophysiology through diverse molecular mechanisms affecting cardiac structure, vascular integrity, and pulmonary circulation (Table 1).

## 9. Limitations and Future Directions

Despite significant progress in understanding m^6^A modifications in obesity, several critical knowledge gaps and methodological limitations persist in current research. First, studies on the role of m^6^A in obesity remain in their nascent stages, with the majority of investigations relying on preclinical models and basic experimental approaches, while translational studies in human populations are notably lacking. Second, while technological advances have improved m^6^A detection capabilities, substantial limitations remain—current methodologies are challenged by the inherent heterogeneity of m^6^A distribution patterns, potentially compromising measurement accuracy and reproducibility. Moreover, existing techniques primarily focus on mRNA modifications, leaving the comprehensive profiling of m^6^A across diverse RNA species (including lncRNAs, circRNAs, and miRNAs) largely incomplete. Most importantly, although accumulating evidence demonstrates robust associations between m^6^A dysregulation and obesity phenotypes, establishing definitive causal relationships remains problematic. A fundamental unanswered question persists regarding whether observed m^6^A alterations constitute secondary consequences of metabolic dysfunction or primary pathogenic drivers—a distinction that demands rigorous investigation through well-designed longitudinal clinical studies and mechanistic experiments.

While m^6^A research has made significant strides in unraveling molecular mechanisms, its clinical translation remains markedly underdeveloped. Future investigations must not only deepen our understanding of m^6^A’s intricate regulatory networks but also prioritize the systematic evaluation of its clinical potential as diagnostic and prognostic biomarkers for obesity and related metabolic disorders. Such translational efforts could yield innovative tools for precision disease management while addressing a critical gap in current research. Notably, the therapeutic targeting of m^6^A modifications remains an untapped clinical frontier, as no m^6^A -directed therapies have yet entered clinical use. This underscores the imperative for pharmacological innovation focused on m^6^A-modifying enzymes, whose strategic manipulation may unlock novel therapeutic paradigms for metabolic diseases. By bridging mechanistic insights with clinical applications, researchers could transform m^6^A biology from a burgeoning field of study into a cornerstone of metabolic disease diagnostics and therapeutics.

As the most prevalent RNA modification, *N*^6^-methyladenosine (m^6^A) methylation serves as a dynamic epigenetic regulator of RNA metabolism, critically influencing RNA stability, splicing, and translational efficiency across diverse biological processes. Notably, the functional consequences of m^6^A modification enzymes exhibit remarkable context-dependent variability across tissue type, developmental stage, or environmental conditions. This review systematically examines the emerging roles of m^6^A modifications in obesity pathogenesis and discusses their translational potential for precision medicine approaches in obesity management. However, current insights into m^6^A’s involvement in metabolic disorders, particularly obesity, remain at a preliminary stage. Future investigations should aim to: (1) comprehensively elucidate the spatiotemporal dynamics of m^6^A -mediated regulation in metabolic tissues, including its interplay with nutrient-sensing pathways and hormonal signaling networks, and (2) rigorously evaluate its clinical potential as both a diagnostic biomarker and novel therapeutic target for obesity intervention strategies. Addressing these priorities will advance our mechanistic understanding of obesity pathophysiology while bridging the translational gap between m^6^A biology and innovative therapeutic interventions for metabolic diseases.

## Figures and Tables

**Table 1:** The regulatory role of m^6^A modification in obesity-related diseases.

Diseases	m^6^A Regulatory Factors	Organ	Expression	Refs.
Type 2 diabetes	METTL14	Brown adipose tissue	Upregulated	[66]
	METTL14	Pancreas	Downregulated	[67]
	METTL3	Liver	Upregulated	[59]
Nonalcoholic fatty liver disease	FTO	Liver	Upregulated	[69]
	METTL14	Liver	Downregulated	[70]
	METTL3	Liver	Downregulated	[70]
Heart disease	ALKBH5	Heart	Upregulated	[72]
	METTL3	Heart	Upregulated	[71]
	FTO	Heart	Downregulated	[73]
Vascular disease	METTL14	Thoracic aorta	Upregulated	[76]
	METTL14	Abdominal aorta	Upregulated	[75]

## Abbreviations

m^6^A	*N*^6^-methyladenosine modification
CVD	cardiovascular diseases
T2DM	type 2 diabetes mellitus
NAFLD	non-alcoholic fatty liver disease
OSA	obstructive sleep apnea
OA	osteoarthritis
NGS	next-generation sequencing
WAT	white adipose tissue
BAT	brown adipose tissue
UCP1	uncoupling protein 1
MSCs	mesenchymal stem cells
METTL3	methyltransferase-like 3
METTL14	methyltransferase-like 14
MTC	m^6^A methyltransferase complex
VIRMA	Vir Like m^6^A Methyltransferase Associated
HAKAI	E3 Ubiquitin-Protein Ligase Hakai
ZC3H13	zinc finger CCCH domain-containing protein 13
RBM15B	RNA Binding Motif Protein 15B
METTL16	methyltransferase-like 16
METTL5	methyltransferase-like 5
ZCCHC4	Zinc Finger CCHC-Type Containing 4
FTO	fat mass and obesity-associated protein
ALKBH5	AlkB homolog 5
WTAP	Wilms tumor 1-associated protein
YTHs	YTH domain-containing proteins
HNRNPs	heterogeneous nuclear ribonucleoproteins
IGF2BPs	IGF2BP family
EIF3	eukaryotic initiation factor 3
FMRP	Fragile X mental retardation protein
PGC1α	peroxisome proliferator-activated receptor γ coactivator 1α
ATG5	Autophagy Related 5
oxLDL	oxidized low-density lipoprotein
DHA	Docosahexaenoic acid
Ddit4	DNA damage-inducible transcript 4
GHRP	growth hormone-releasing peptide
AAA	Abdominal aortic aneurysm
